# Is the trend toward a sustainable green synthesis of copper oxide nanoparticles completely safe for *Oreochromis niloticus* when compared to chemical ones?: using oxidative stress, bioaccumulation, and histological biomarkers

**DOI:** 10.1007/s11356-023-31707-x

**Published:** 2024-01-08

**Authors:** Shereen R. Badran, Aliaa Hamed

**Affiliations:** 1 Department of Zoology, Faculty of Science, Cairo University, Giza, Egypt; 2 Department of Biology, Basic Science Center, Misr University for Science and Technology (MUST), Giza, Egypt

**Keywords:** Environmental sustainability, Nanotoxicology, Copper oxide nanoparticles, Green synthesis, *Oreochromis niloticus*, Oxidative stress, Bioaccumulation, Histology

## Abstract

Scientists worldwide have noticed that cutting-edge technologies can be used to produce nanoparticles (NPs) in a sustainable and environmentally friendly way, instead of the old methods. However, the effectiveness of this approach for aquatic environments and species still needs to be determined. Therefore, this study aims to compare between the toxicity of green and chemically synthesized copper oxide nanoparticles (GS and CS) CuO NPs at two different concentrations on Nile tilapia (*Oreochromis niloticus*) using various biomarkers. CuO NPs’ formation was proved, and their different characterizations were recorded. Then, the fish samples were randomly allocated in glass aquaria into five groups: one acted as a control group, and the other groups were exposed to two concentrations (25 and 50 mg/L) of GS-CuO NPs and CS-CuO NPs, separately, for 4 days. After the experimental time, in all groups that were exposed to two concentrations of both synthesized CuO NPs, the results revealed that glutathione peroxidase (GPx), catalase (CAT), superoxide dismutase (SOD), and thiobarbituric acid reactive substances (TBARS) levels were elevated in the liver and gills compared to glutathione reduced (GSH) content, which showed a significant decline. Bioaccumulation of Cu was more prevalent in the liver than in the gills, and the highest bioaccumulation capacity was more evident in the groups exposed to CS-CuO NPs. Moreover, the bioaccumulation of Cu caused severe histological changes in the liver and gills. In conclusion, the results suggested that GS-CuO NPs revealed less toxicity than CS-CuO NPs to the examined fish. However, they are still toxic, and their toxic effect cannot be overlooked.

## Introduction

Nanotechnology has rapidly evolved and spread worldwide in recent years; it has several applications in medicine, agriculture, food production, and aquaculture (Rahman et al. 2022). In addition, metal oxide NPs have garnered much attention because of their typical physicochemical features (Aziz and Abdullah 2023). CuO NPs, one metal oxide NPs, have been produced more due to their excellent catalytic, antibacterial, anticancer, and thermo-physical properties (Prasad et al. 2016; Roy et al. 2021). They are commonly used in chemical processes, solar cells, gas sensors, lithium batteries, electronics, catalysts, nutrient protection, textile industries, coatings, drug delivery, wound dressings, wastewater treatment, agriculture, and face masks (Wu et al. 2020; Manjula et al. 2022). Thus, they are regarded more abundant in aquatic environments than other NPs due to their extensive use and lower cost over the last several decades (Forouhar Vajargah et al. 2020). To produce CuO NPs, various techniques, including physical, chemical, and green ones, are available (Heikal et al. 2020). Physical and chemical procedures are costly and time-consuming; they also necessitate high energy and the use of hazardous and extremely toxic substances (Jafari et al. 2022). On the other hand, the domains of nanotechnology are increasingly concerned with sustainability strategies to lessen the adverse environmental impacts (Falinski et al. 2018; Abdelbasir et al. 2020; Auclair et al. 2023). Therefore, the green synthesis method has been prioritized since it is eco-friendly and concerned with sustainability plans more than other methods (Ahamed et al. 2022). Due to the potent phytochemicals that function as reducing agents, such as ascorbic acids, carboxylic acids, phenols, amides, sugars, aldehydes, and flavonoids, many plants have been widely used in green synthesis (Perveen et al. 2020). CuO NPs are extremely dangerous to the environment and aquatic life because they remain in the environment and food chains for a long time (Rashidian et al. 2021). Previous research has shown that CuO NPs are harmful to many fish. For example, they have been reported to affect the antioxidant systems of zebrafish (*Danio rerio*), African catfish (*Clarias gariepinus*), and *O. niloticus* (Ganesan et al. 2016; Tuncsoy et al. 2017; Canli and Canli 2020). Moreover, they can build up in the tissues of *C. gariepinus* (Duran et al. 2017). Exposure to CuO NPs also causes significant histological changes in the organs of rainbow trout (*Oncorhynchus mykiss*), guppy (*Poecilia reticulata*), common carp (*Cyprinus carpio*), and streaked prochilod (*Prochilodus lineatus*) species (Al-Bairuty et al. 2013; Mansouri et al. 2015; Vajargah et al. 2018; Tesser et al. 2020). Consequently, it is necessary to evaluate their toxicity in the aquatic habitat and its biota. Because of its long-term tolerance to several aquatic stresses, *O. niloticus* has been used as animal model in many toxicological studies (Ibrahim et al. 2021). Additionally, various biomarkers have been frequently used to assess NPs toxicity in aquatic species (Kaviani et al. 2019; Abdel-Khalek et al. 2020a; Mahjoubian et al. 2023b). One promising biomarker, for instance, was the change in antioxidant status (Ozmen et al. 2020; García-Medina et al. 2022). Furthermore, bioaccumulation potential and histological change assessment can provide alerts for synthesized CuO NPs toxicity on various fish tissues, and their whole health conditions (Abdel-Khalek et al. 2016). Although much research has focused on synthesizing CuO NPs using chemical and green procedures, few data are available on the comparative toxicity of GS and CS CuO NPs in fish (Nasrullah et al. 2020; Sabeena et al. 2022). Therefore, our study aims to examine the toxicity of GS and CS CuO NPs, with two sublethal concentrations for each, and evaluate their toxic effects on the most common freshwater fish, *O. niloticus*. In addition, it investigates whether GS-CuO NPs are eco-friendly or not safe for the aquatic environment, such as those synthesized by chemical methods.

## Materials and methods

### Chemicals

Copper II acetate monohydrate (Cu(C_2_H_3_O_2_)_2_ H_2_O), and glacial acetic acid (CH_3_COOH) were bought from El Nasr Pharm. Chem. Company, Egypt. Moreover, sodium hydroxide (NaOH) was obtained from Techno Pharmchem, India. All purchased chemicals were of analytical grade.

### Green and chemical CuO NP synthesis

CuO NPs were synthesized according to Nwanya et al. (2019) for green synthesis and Zhu et al. (2011) for chemical synthesis, with minor modifications. To prepare GS-CuO NPs, fresh maize (*Zea maize* L.) husks were acquired from local merchants in Giza, Egypt. They were sun-dried until they became friable for grounding into powder. Then, 10 gr of powder was dissolved in 200 mL of deionized water and boiled at 60 °C for 2 h until the extract became a faint yellow. Next, the extract was cooled to room temperature. After that, it was filtered three times with Whatman filter paper. Finally, 2 gr of Cu(C_2_H_3_O_2_)_2_ H_2_O was dissolved in 50 mL of the extract; and after 2 h of heating, the black precipitate appeared in the flask bottom. In addition, to prepare CS-CuO NPs, 2 gr Cu(C_2_H_3_O_2_)_2_ H_2_O was dissolved in 50-mL deionized water. Then, 0.2 mL CH_3_COOH was added to the solution and heated with continuous stirring. After 30 min, 2.5 mL of 8 M NaOH was slowly added into the flask with continuous stirring for 2 h to produce the black precipitate. Furthermore, the precipitates from these two methods were centrifuged numerous times and washed with deionized water to eliminate any leftover aqueous extract and remove any impurities. After that, the purified precipitates were dried at 60 °C overnight and calcined for 4 h at 300 and 600 °C in the muffle furnace. Finally, the black-produced CuO NPs powders were collected and stored for later analysis.

### Synthesized CuO NP characterization

The synthesized CuO NPs were subjected to several techniques for various characterizations, according to Yugandhar et al. (2017), Joshi et al. (2019), Sharma et al. (2019), Akintelu et al. (2020), and Chandrasekaran et al. (2020). CuO NPs’ formation was confirmed using a UV–vis spectrophotometer (UV-1800, Shimadzu, Japan) in the 200–800-nm wavelength range. In addition, morphology and size were analyzed by field emission transmission electron microscopy (FETEM, JEM-2100F, JEOL Inc., Japan), where one drop of NPs suspension was applied on a carbon-coated copper grid, air-dried. Then, the surface analysis was performed at a voltage of 200 kV (Akhtar et al. 2016). Simultaneously, selected area electron diffraction (SAED) analysis was carried out on the TEM device to evaluate crystallinity of the sample by using an ImageJ software tool to determine the d-spacing referred to the theta and hkl integers, as reported by Barua et al. (2019) and Das et al. (2022). Furthermore, to verify the elemental composition of CuO NPs, scanning electron microscopy (FEI-SEM, Inspect S50, Netherlands) was conducted at a 15–30 kV voltage equipped with an energy dispersive X-ray spectrophotometer (EDX). As described by Murdock et al. (2008), the zeta potential and dynamic light scattering (DLS) (Nano-Zeta Sizer-HT, Malvern Instruments, UK) analyses were also used to assess the CuO NP stability and average hydrodynamic size in water, respectively.

### Acclimatization of the examined fish

The adult male fish were bought from a clean ranch in Kafr El-Sheikh, Egypt and transported in well-aerated tanks to the ecology lab at the Faculty of Science, Cairo University. Before the beginning of the experiment, fish were distributed randomly in dechlorinated water glass aquaria (50 L) having dimensions of 40 × 70 × 26 cm with continuous aeration for a 10-day adaptation period. Water conditions were as follows: 24 ± 1 °C for temperature, 7.2–7.4 for pH, and the dissolved oxygen concentration was between 6.6 and 7.9 mg/L. The water was changed daily (30% of it) to remove excreta and excess food in the glass aquarium. Additionally, fish were fed commercial pellet meal daily (3% of body weight), which contained 20% crude protein, 5% crude fiber, 4% crude fat, 10% crude moisture, and 13% crude ash. They were fed until reaching apparent satiation (NRC 1993).

### Exposure stock suspension preparation of CuO NPs

The stock suspension of CuO NPs was prepared in low-density polyethylene bottles (to prevent CuO NPs glass sticking) by dispersing NPs and stirring them with a magnetic stirrer (IKA Werke RET basic C) at 300 rpm in deionized water for 1 h, as suggested by Al-Bairuty et al. (2016). Before adding CuO NPs to the water in the fish aquaria, the stock suspension was diluted to 25 mg/L and 50 mg/L, the concentrations under investigation, with dechlorinated tap water, and stirred for 20 min to avoid aggregation.

### Experimental setup

After the adaptation time, fifty fish (40 ± 2.5 gr and 13 ± 0.5 cm) were randomly allocated in 50 L of well-aerated glass aquaria (5 fish/aquarium) into five groups with duplicated aquaria per group. One of these groups acted as the control group with dechlorinated water only. The other groups were exposed to two concentrations (25 and 50 mg/L) of GS-CuO NPs and CS-CuO NPs, separately, for 4 days. Both concentrations were chosen as sub-lethal to *O. niloticus*, according to Abdel-Latif et al. (2021), who found that the LC_50_ value was 100 mg/L. During the experiment, water was changed daily (30%) and re-dosed with a new aliquot of CuO NPs to maintain the exposure at relatively consistent levels. The water conditions were also regularly checked to ensure that they are similar to those observed throughout the acclimatization phase.

### Fish and tissue sampling

At the end of the experiment time period, the fish were collected from all groups and anesthetized with clove oil (6–17 mg/L, once and by immersion) until opercular movement stopped to ensure euthanasia (Underwood and Anthony 2020). Next, the liver and gills were removed from the dissected fish for later investigations.

### Determination of antioxidant biomarkers

All antioxidant biomarker determination and tissue homogenization were conducted according to the kit's instructions (Biodiagnostic, Dokki, Giza, Egypt). First, the tissues were washed with 0.9% cold NaCl. Second, after homogenizing 1 gr of the tissues (for each kit analysis) in a glass homogenizer containing 5 mL of cold buffer, each underwent cooled centrifugation (4000 rpm, 15 min). Third, the resultant supernatants were carefully collected and stored at – 80 °C to evaluate all the oxidative stress indicators later.

#### Glutathione peroxidase (GPx)

According to Paglia and Valentine (1967), the GPx activity was indirectly measured by adding 10µ of the sample to the solution containing 100 µ (glutathione, glutathione reductase, and NADPH) along with 100 µ hydrogen peroxide (H_2_O_2_). In this reaction, the oxidized glutathione was produced by GPx reduction and then returned to its reduced form by glutathione reductase. The oxidation of NADPH to NADP^+^ that occurred in the reaction with a decline in absorbance per min at 340 nm was related to GPx activity.

#### Catalase (CAT)

Aebi (1984) provided a detailed estimate of CAT activity. In this process, 50 µ of the sample was added to the solution containing 500 µ phosphate buffer and 100 µ H_2_O_2_, in which CAT reacts with H_2_O_2_, and the response was stopped after one min by adding 200 µ catalase inhibitor. Then, the residues of H_2_O_2_ interacted with 3,5-dichloro-2-hydroxybenzene sulfonic acid and 4-aminophenazone after their addition (500 µ) to the reaction solution in the presence of peroxidase to generate a chromophore with a color intensity measured at 510-nm absorbance that is inversely related to the amount of CAT in the sample.

#### Superoxide dismutase (SOD)

The nitroblue tetrazolium dye reduction by the phenazine methosulfate was the base of this assay (Nishikimi et al. 1972). Then, 100 µ of the sample was mixed well with 1 mL of working solution (phosphate buffer, nitroblue tetrazolium, and NADH). Then, the reaction was initiated by adding 100µ phenazine methosulphate (PMS). After that, the SOD activity (inhibition rate) was measured for a 5-min increase in the absorbance at 560 nm.

#### Lipid peroxide (LPO)

The levels of TBARS were a significant measure for determining lipid peroxidation. According to Ohkawa et al. (1979), TBARS resulted from the 30-min reaction of 1 mL of thiobarbituric acid with malondialdehyde in the sample (200 µ) at 95 °C. The strength of the resultant pink color related to TBARS quantities in the samples was spectrophotometrically translated to absorbance at 534 nm.

#### Glutathione reduced (GSH)

Beutler et al. (1963) stated that GSH determination was done by mixing the 100-µ sample with 500 µ trichloroacetic acid, leaving it for 5 min at room temperature, and then centrifuging it at 3000 rpm for 15 min. Moreover, 500 µ of the resultant supernatant was mixed with 1-mL buffer. Next, the reduction occurred by adding 500 µ of 5, 5′-dithiobis (2-nitrobenzoic acid), which produced a yellowish color that was determined at 405-nm absorbance.

### Copper bioaccumulation

Inductively coupled plasma atomic emission spectroscopy (Thermo Scientific, iCAP 6000 series model) was used to assess fish liver and gill copper bioaccumulation (mg/kg) via the dry ashing procedure (APHA 2005). After drying the tissues in the oven at 80 °C for 8 h, they were acid digested with 3 mL of concentrated hydrochloric acid (HCl). Then, the resultant was diluted to 25 mL with deionized water. Procedure blanks were conducted throughout the testing to account for background absorption, and to verify the accuracy of the measurement method. The analytical method was validated using standard reference material from the National Institute of Standards and Technology (USA) (Lake Superior Fish 1946). Cu-metal recoveries were 95–110%. Furthermore, the bioaccumulation factors (BAFs) of synthesized NPs were estimated, as stated by Authman and Abbas (2007): BAF equals the concentration of Cu in tissues (mg/kg)/their concentration in water (mg/L).

### Histological study

After being isolated, the liver and gills were washed with NaCl (0.7%) and then well-maintained in Bouin’s fixative. After fixation, the tissues were dehydrated, embedded in paraffin wax, and underwent 4-μm sections using a microtome. Finally, the tissues were stained with hematoxylin and eosin. The procedures were carried out based on Suvarna et al. (2018). Besides, light microscopy was used to record the histological changes in the tissue specimens of each group examined.

### Statistical analysis

The independent sample *t*-test assessed the difference between the two groups exposed to synthesized CuO NPs, with regard to each concentration. Additionally, one-way variance analysis (ANOVA) was used to assess the variation of the studied concentrations for each group that received synthesized CuO NPs, in comparison to the control group. Duncan’s multiple range test was also utilized for similarity measurements within the different groups, denoted by various upper and lower cases in decreasing order. If *p* < 0.05, the mean difference was considered significant, and all results were reported as mean ± SE (standard error). The statistical analysis was done using SPSS 16.0 statistical software (IBM, Chicago, IL).

## Results

### Synthesized CuO NP characterization

**Fig. 1:**
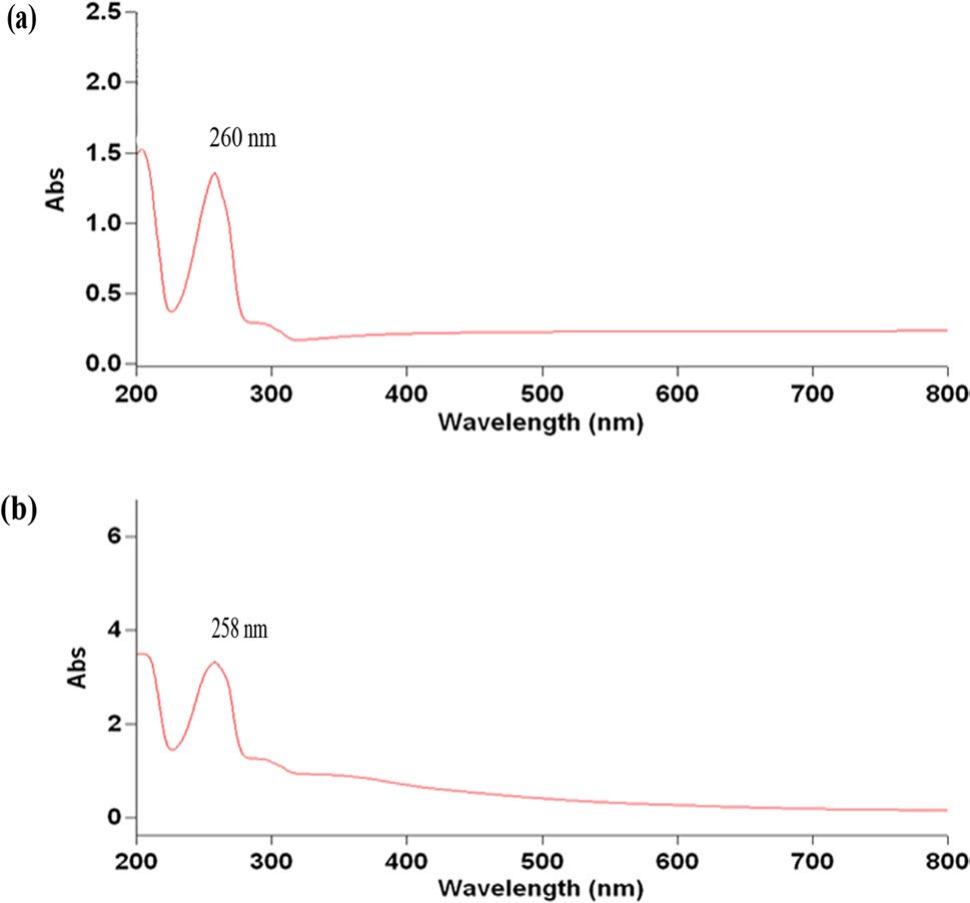
Representative UV–visible absorbance spectra of **a** GS-CuO NPs and **b** CS-CuO NPs

**Fig. 2:**
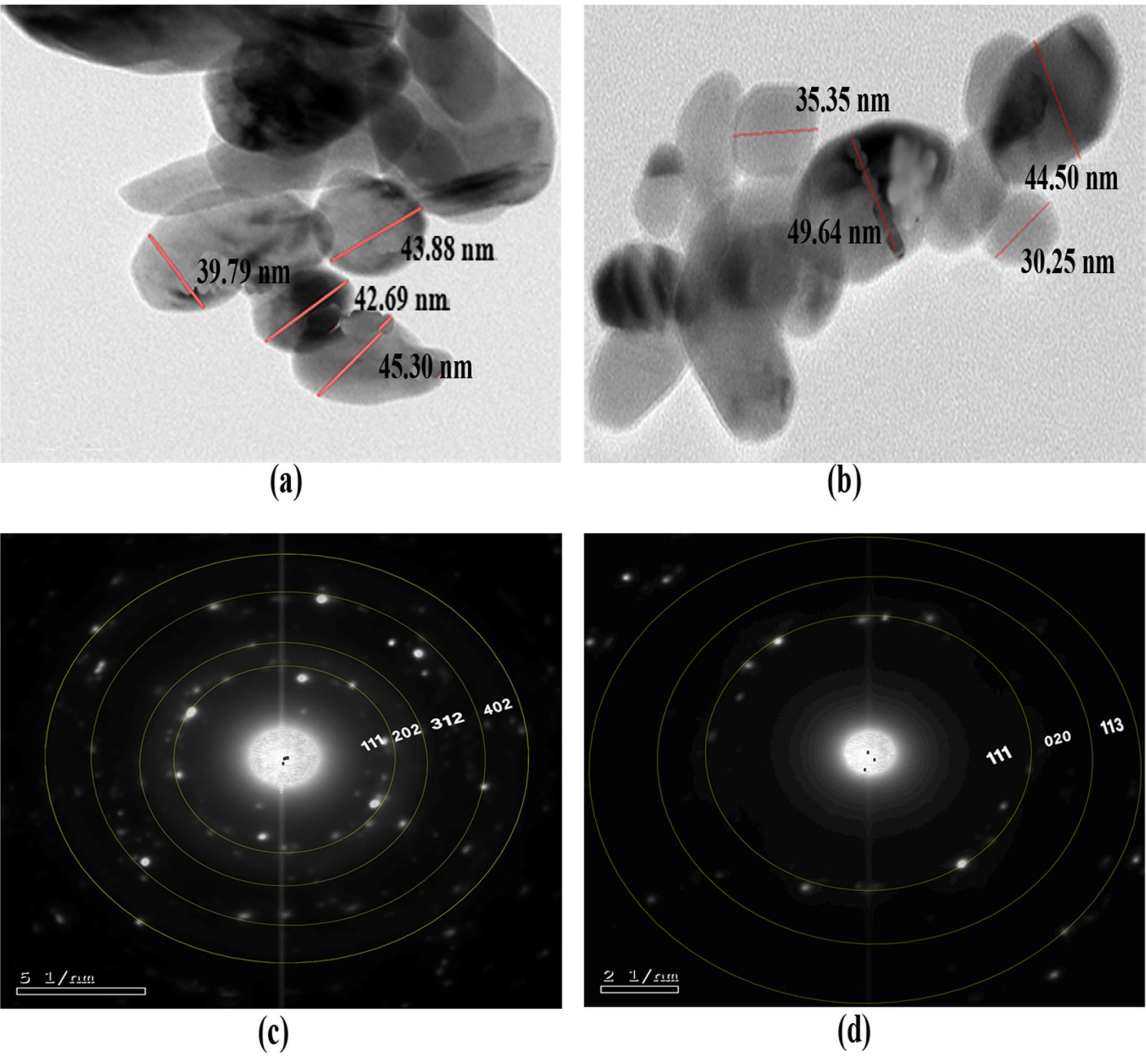
Transmission electron microscopy (TEM) images of **a** GS-CuO NPs and **b** CS-CuO NPs and selected area diffraction (SAED) profiles of **c** GS-CuO NPs and **d** CS-CuO NPs

**Fig. 3:**
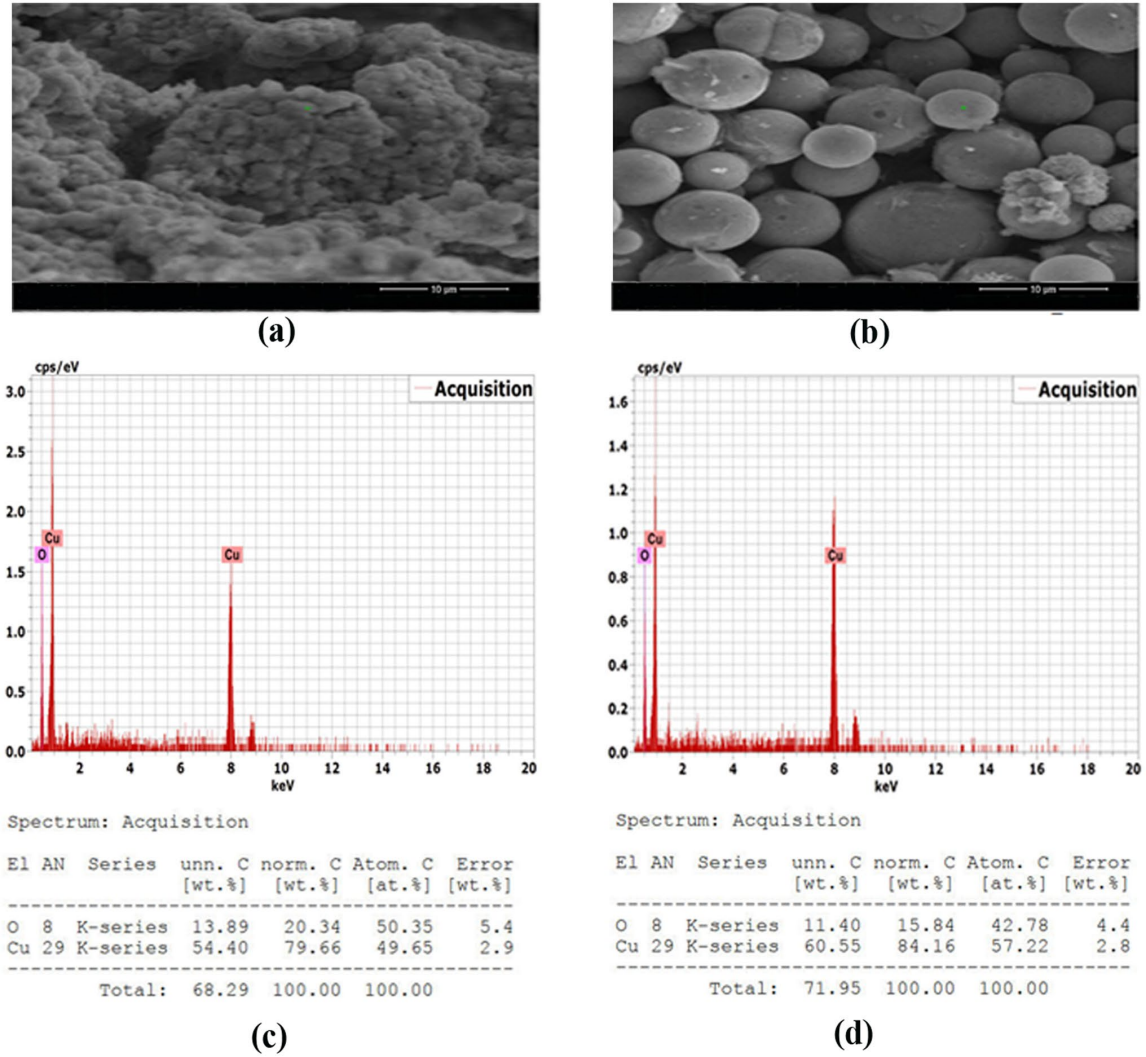
Scanning electron microscope (SEM) images of **a** GS-CuO NPs and **b** CS-CuO NPs and energy-dispersive X-ray spectroscopy (EDX) spectra of **c** GS-CuO NPs and **d** CS-CuO NPs

**Fig. 4:**
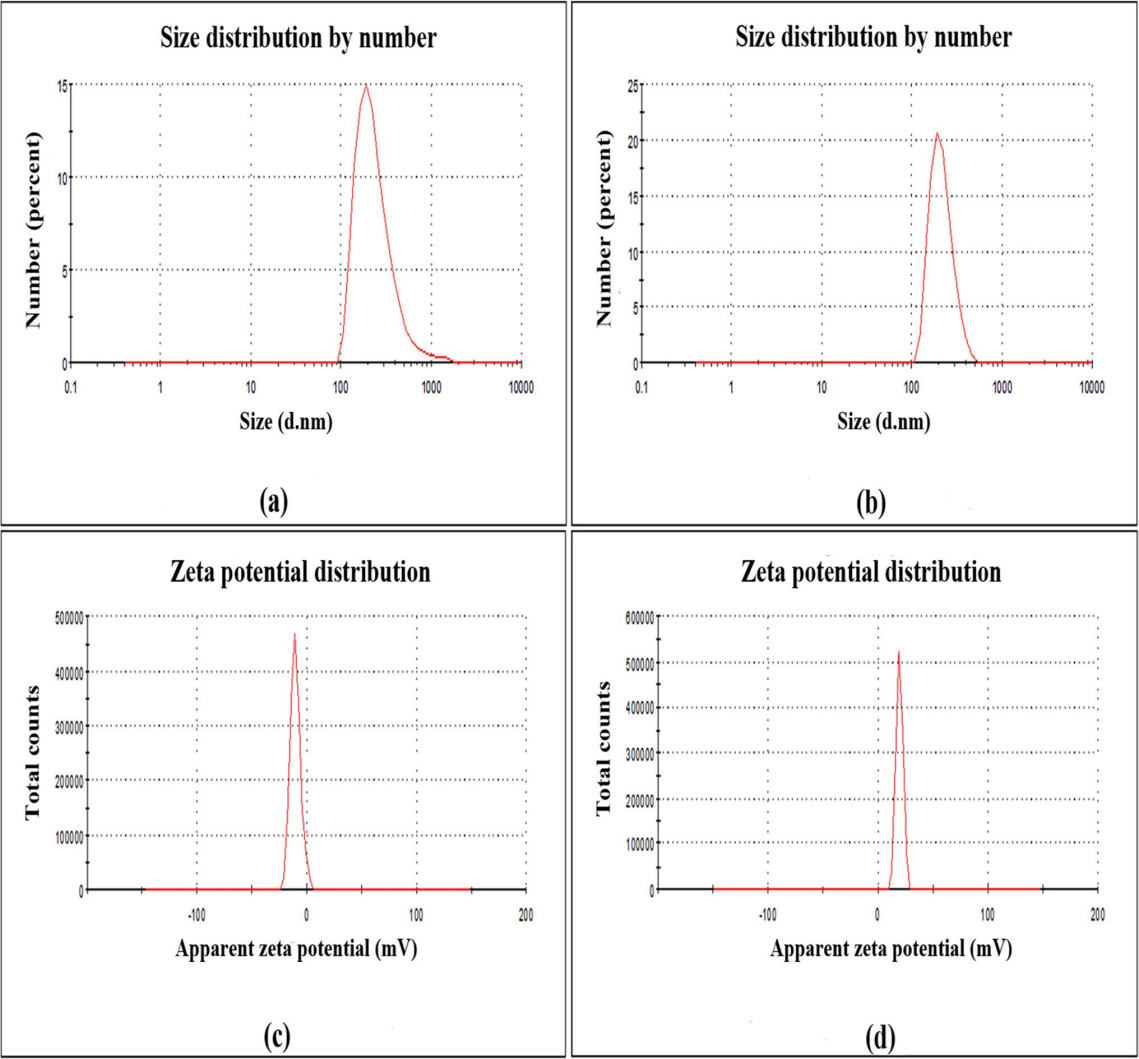
The average hydrodynamic sizes (DLS) of **a** GS-CuO NPs and **b** CS-CuO NPs and zeta potential distributions of **c** GS-CuO NPs and **d** CS-CuO NPs

The acquired CuO NPs were described via UV–vis spectrophotometer, TEM, SAED, SEM, EDX, DLS, and zeta potential (Figs. 1, 2, 3, and 4). The GS-CuO NP spectra, with the peaks slightly shifted, showed an absorption wavelength of 260 nm (Fig. 1a) and that of CS-CuO NPs was 258 nm (Fig. 1b). TEM images (Fig. 2a, b) also displayed that the synthesized CuO NPs were spherical, < 50 nm. CuO NPs SAED profiles (Fig. 2c, d) revealed the presence of bright circular spots with diffraction patterns directed towards (111), (202), (312), and (402) planes for GS-CuO NPs and directed toward (111), (020), and (113) planes for CS-CuO NPs, which is in accordance with Diffraction Data (ICDD) card no. 00–041-0254. Moreover, SEM and EDX were utilized to study the morphological surface and compositional information features of the synthesized CuO NPs. The particles examined by SEM images were relatively spherical and suggested that GS-CuO NPs were more aggregated than CS-CuO NPs (Fig. 3a, b). EDX spectra (Fig. 3c, d) showed that the synthesized CuO NPs were highly pure, as Cu and O were the main elements; and no irrelevant peaks were detected. For GS-CuO and CS-CuO NPs, the average hydrodynamic sizes (DLS) in water were 259.4 nm and 218.3 nm, respectively (Fig. 4a, b); additionally, the zeta potentials were − 10 mV and 19.5 mV, respectively (Fig. 4c, d).

### Oxidative stress biomarkers

**Fig. 5:**
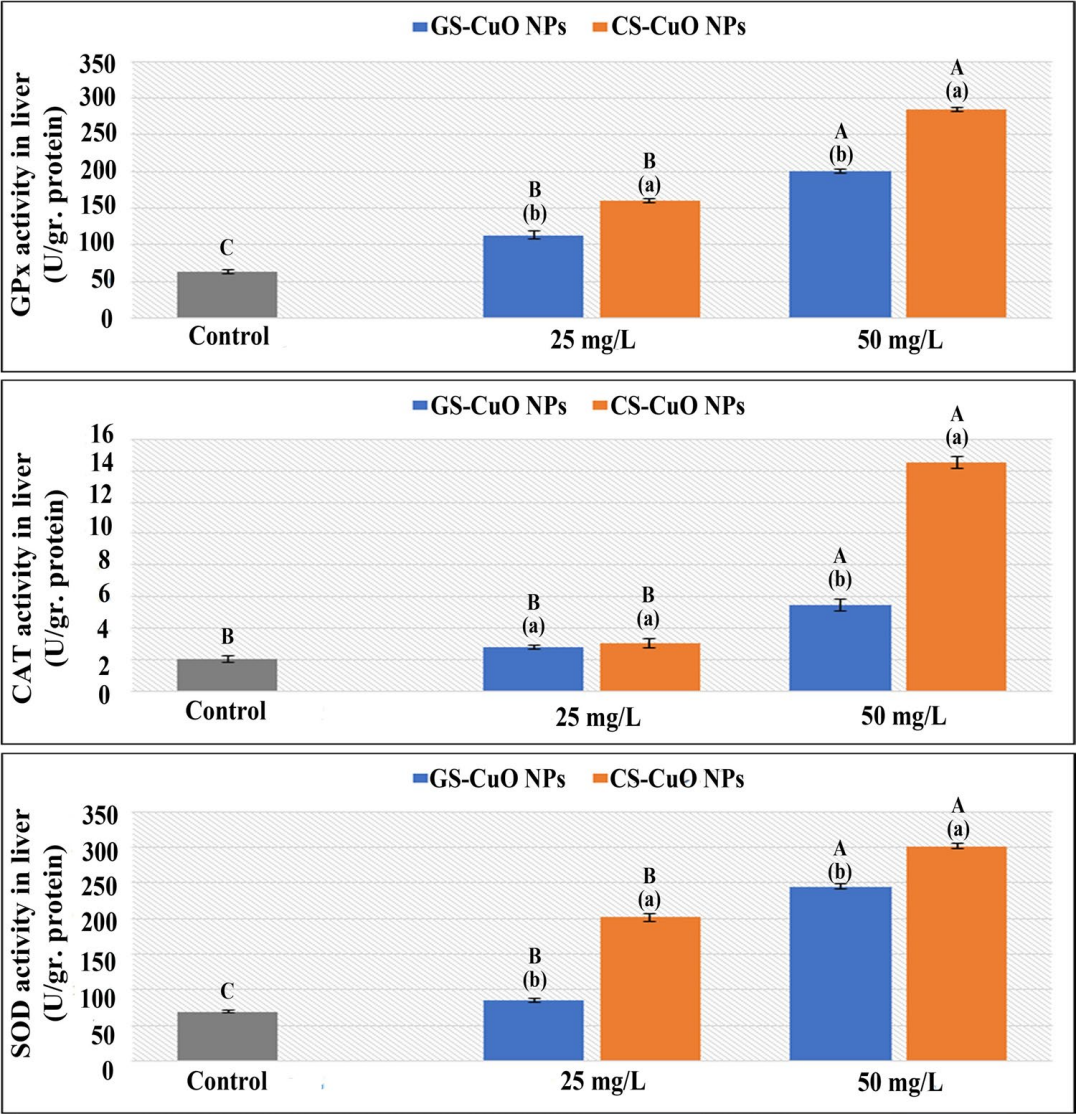
Changes in the liver GPx, CAT, and SOD of *O. niloticus* groups exposed to two different concentrations (25 and 50 mg/L) of GS-CuO NPs and CS-CuO NPs. Results are indicated as means of 8 fish per group ± SE. The different lowercase (*p* < 0.05) shows the significant difference between the groups exposed to GS and CS CuO NPs for each concentration. The different uppercase (*p* < 0.05) indicates a significant difference among the 25 and 50 mg/L groups for each synthesized CuO NPs, compared with the control group. Columns with identical letters do not noticeably differ from the others

**Fig. 6:**
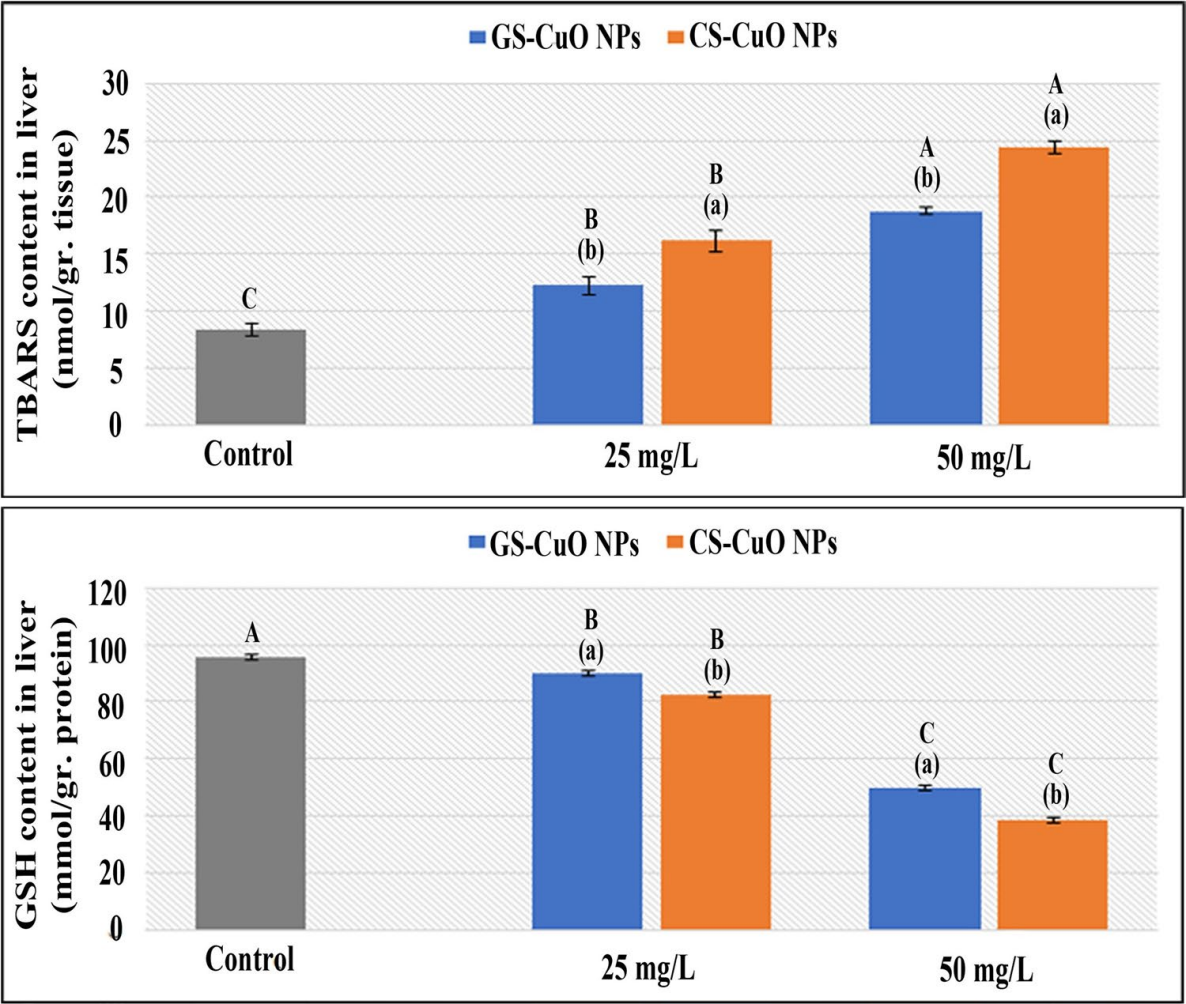
Changes in the liver TBARS and GSH of *O. niloticus* groups exposed to two different concentrations (25 and 50 mg/L) of GS-CuO NPs and CS-CuO NPs. Results are indicated as means of 8 fish per group ± SE. The different lowercase (*p* < 0.05) shows the significant difference between the groups exposed to GS and CS CuO NPs for each concentration. The different uppercase (*p* < 0.05) indicates a significant difference among the 25 and 50 mg/L groups for each synthesized CuO NPs, compared with the control group. Columns with identical letters do not noticeably differ from the others

**Fig. 7:**
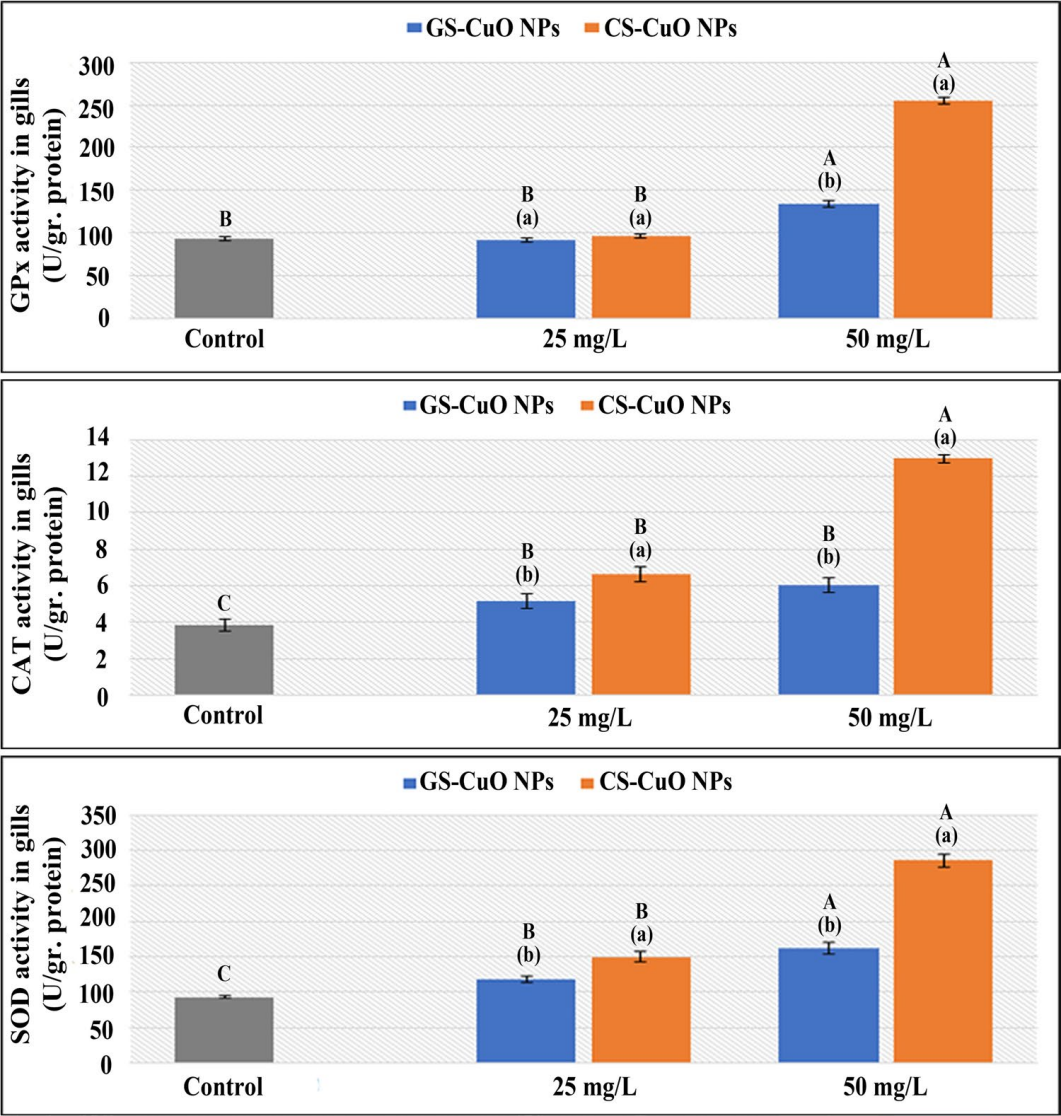
Changes in the gills GPx, CAT, and SOD of *O. niloticus* groups exposed to two different concentrations (25 and 50 mg/L) of GS-CuO NPs and CS-CuO NPs. Results are indicated as means of 8 fish per group ± SE. The different lowercase (*p* < 0.05) shows the significant difference between the groups exposed to GS and CS CuO NPs for each concentration. The different uppercase (*p* < 0.05) indicates a significant difference among the 25 and 50 mg/L groups for each synthesized CuO NPs, compared with the control group. Columns with identical letters do not noticeably differ from the others

**Fig. 8:**
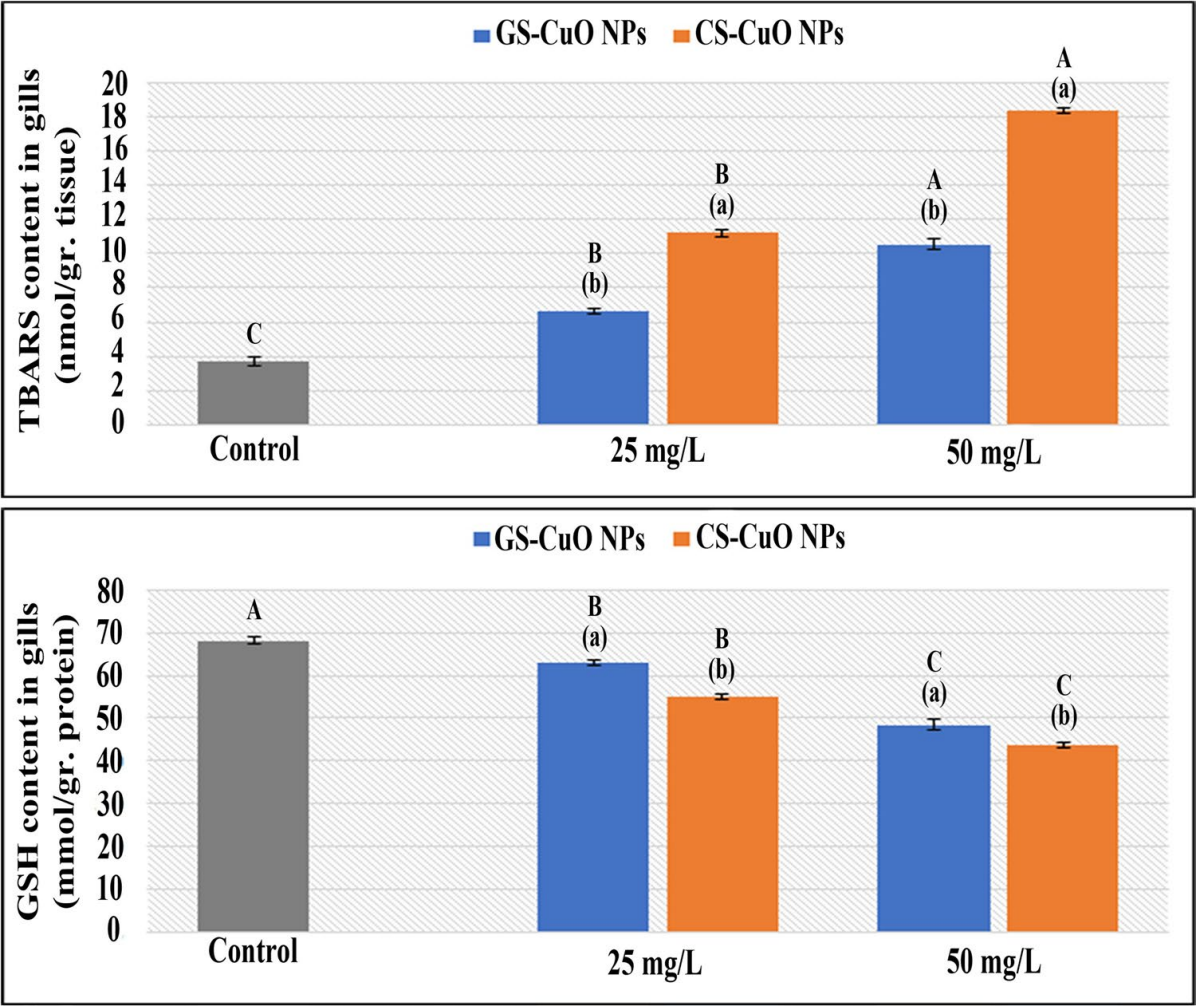
Changes in the gills TBARS and GSH of *O. niloticus* groups exposed to two different concentrations (25 and 50 mg/L) of GS-CuO NPs and CS-CuO NPs. Results are indicated as means of 8 fish per group ± SE. The different lowercase (*p* < 0.05) shows the significant difference between the groups exposed to GS and CS CuO NPs for each concentration. The different uppercase (*p* < 0.05) indicates a significant difference among the 25 and 50 mg/L groups for each synthesized CuO NPs, compared with the control group. Columns with identical letters do not noticeably differ from the others

The effects of synthesized CuO NPs at 25 and 50 mg/L showed a substantial increase in liver enzymatic biomarkers and TBARS. In contrast, a significant GSH content decrease was detected (Figs. 5 and 6). All gill oxidative test indicators (Figs. 7 and 8) showed the same path of toxic effects, such as in the liver. At both doses, but particularly with high concentration (50 mg/L), all biomarkers in the liver and gills displayed that the chemically synthesized ones were the most harmful.

### Copper bioaccumulation

The bioaccumulation capacities of Cu and BAFs in the liver and gills of *O. niloticus* were recorded in Table 1. In terms of Cu bioaccumulation in the liver and gills, there was a significant increase after 25 and 50 mg/L of (GS and CS) CuO NP exposure, with the maximum elevation in the groups that received 50 mg/L of CS-CuO NPs. Furthermore, the findings revealed that the liver, rather than the gills, had the highest bioaccumulation capacities for Cu. When BAFs in the liver and gills were compared, the results indicated that CS-CuO NPs had a greater entrance percentage than GS-CuO NPs, especially in the 25 mg/L group.

**Table 1:** The bioaccumulation capacities of Cu and bioaccumulation factors (BAFs) in the liver and gills of *O. niloticus* exposed to two different concentrations (25 and 50 mg/L) of GS-CuO NPs and CS-CuO NPs. Results are indicated as means of 8 fish per group ± SE. The different lowercase (*p* < 0.05) shows the significant difference between the groups exposed to GS and CS CuO NPs in each concentration. The different uppercase (*p* < 0.05) displays a significant difference among the 25 and 50 mg/L groups for each synthesized CuO NPs, compared with the control group. Rows and columns with identical letters do not noticeably differ from the others. NS: not significant

	Liver					Gills				
	GS-CuO NPs		CS-CuO NPs			GS-CuO NPs		CS-CuO NPs		
	Accumulated Cu (mg/kg dry weight)	BAFs	Accumulated Cu (mg/kg dry weight)	BAFs	*P*_t_	Accumulated Cu (mg/kg dry weight)	BAFs	Accumulated Cu (mg/kg dry weight)	BAFs	*P*_t_
Control	**167.80**^**a**^** ± 4.73**^**C**^	–	**167.80**^**a**^** ± 4.73**^**C**^	–		**73.60**^**a**^** ± 3.03C**	–	**73.60**^**a**^** ± 3.03C**	–	**NS**
25 (mg/L)	**204.69**^**b**^** ± 10.97**^**B**^	**8.19**	**433.70**^**a**^** ± 11.07B**	**17.35**		**168.05**^**b**^** ± 5.21B**	**6.72**	**198.60**^**a**^** ± 7.32**^**B**^	**7.94**	** < 0.05**
50 (mg/L)	**223.72**^**b**^** ± 12.05**^**B**^	**4.47**	**596.97**^**a**^** ± 11.70**^**A**^	**11.94**		**287.13**^**b**^** ± 8.30A**	**5.74**	**375.13**^**a**^** ± 11.67A**	**7.50**	** < 0.05**
*P*_f_	** < 0.05**	–	** < 0.05**	–		** < 0.05**	–	** < 0.05**	–	–

### Histological study

**Fig. 9:**
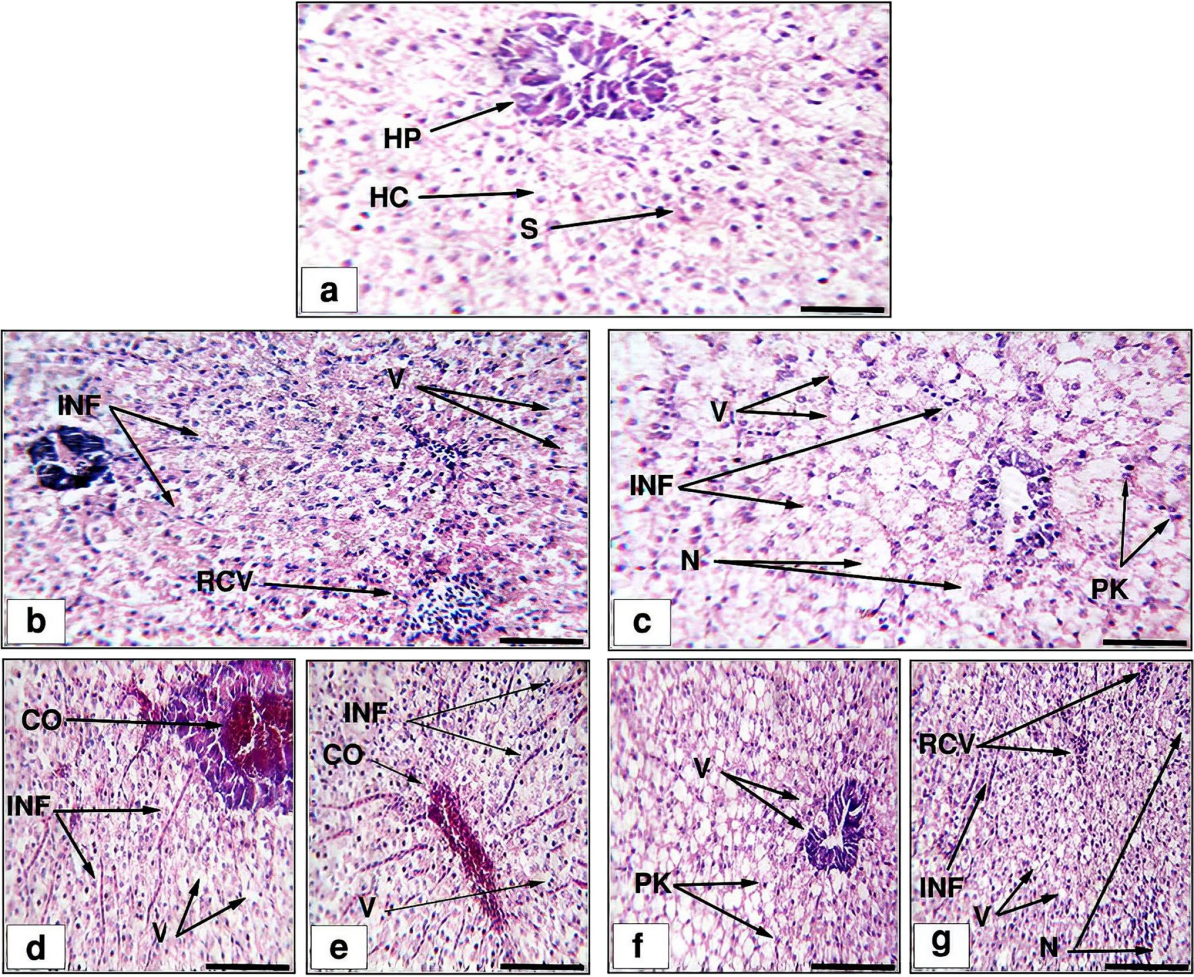
Representative histological liver alterations of *O. niloticus* (scale bar = 100 μm). **a** The control group, **b** 25 mg/L GS-CuO NPs exposed group, **c** 25 mg/L CS-CuO NPs exposed group, **d, e** 50 mg/L GS-CuO NPs exposed group, and **f, g** 50 mg/L CS-CuO NPs exposed group. HC, hepatic cells; HP, hepatopancreas; S, sinusoids; RCV, rupture of central vein; V, cytoplasmic vacuolation; INF, infiltration of blood cells; PK, pyknotic nuclei; N, necrosis; CO, congestion in blood vessels

**Fig. 10:**
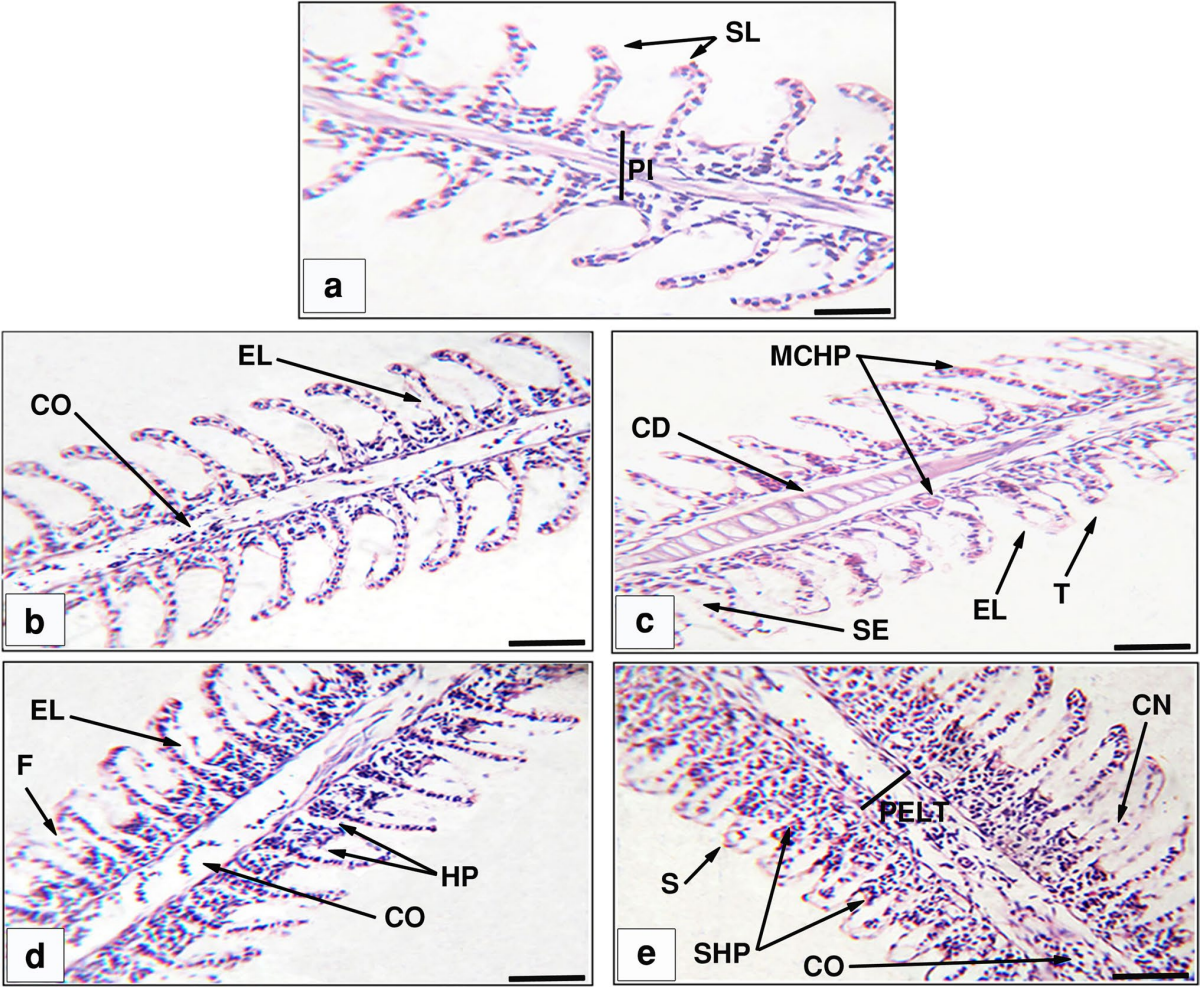
Representative histological gill alterations of *O. niloticus* (scale bar = 100 μm). **a** The control group, **b** 25 mg/L GS-CuO NPs exposed group, **c** 25 mg/L CS-CuO NPs exposed group, **d** 50 mg/L GS-CuO NPs exposed group, and **e** 50 mg/L CS-CuO NPs exposed group. PL, primary lamellae; SL, secondary lamellae; EL, epithelial lifting; HP, hyperplasia; CD, cartilage deformation; SE, severe edema; T, telangiectases at the tip of secondary lamellae; F, fusion in secondary lamellae; MCHP, mucosal cell hyperplasia; CO, congestion in the lamellar blood vessels; S, shortening to secondary lamellae; SHP, severe hyperplasia; PLET, primary lamellar epithelial thickening; CN, cellular necrosis

The 25 and 50 mg/L toxic effects of the studied synthesized NPs on the histological structures of *O. niloticus* liver and gills are shown in Figs. 9 and 10. The liver tissue of the control group (Fig. 9a) displayed a normal structure, with sinusoids (S) dispersed randomly throughout the hepatocytes and densely organized polygonal hepatocytes (HC), revealing cytoplasm with sphere nuclei. The histologic alterations in the fish liver tissues exposed to sub-lethal doses of CuO NPs (Fig. 9b–g) manifested blood cell infiltration (INF), rupture of the central vein (RCV), cytoplasmic vacuolization (V), pyknotic nuclei (PK), necrosis (N), and congestion in the blood vessels (CO). Besides, the gills in Fig. 10a showed well-structured gill filaments with primary and secondary lamellas and flat epithelial cells. The gills' histological alterations, including blood vessel congestion (CO), epithelial lifting (EL) at secondary lamellae, cartilage deformation (CD), severe edema (SE), telangiectases (T), and mucosal cell hyperplasia (MCHP), were recorded in the groups that received the 25 mg/L concentration (Fig. 10b, c). On the other hand, the gill sections obtained from the 50 mg/L groups showed fusion in the secondary lamellae (F), primary lamellae epithelial hyperplasia (HP), shortening to secondary lamellae (S), severe hyperplasia (SHP), primary lamellar epithelial thickening (PLET), cellular necrosis (CN), and congestion in the lamellar blood vessels. The findings also revealed that the groups that were exposed to CS-CuO NPs had the most visible histological alterations in the studied tissues. Moreover, the severity of the alterations was dose dependent.

## Discussion

CS-CuO NPs are cause for concern because they use highly toxic chemicals released into aquatic environments and harm aquatic animals. GS-CuO NPs are said to be eco-friendly, but that does not mean they are thoroughly safe for aquatic animals (Ibrahim et al. 2021). Thus, the present study made a comparison between GS-CuO NPs and CS-CuO NPs based on their synthesis, nature in water, and their possible toxic effects on various health biomarkers (oxidative stress, bioaccumulation of Cu, and histological changes) on the liver and gills of *O. niloticus*.

### Synthesized CuO NP characterization

The reduction of copper II acetate monohydrate to CuO NPs was first physically verified by a color change to black powder, as mentioned in previous studies (Nagaraj et al. 2019; Awwad and Amer 2020; Fakhar-e-Alam et al. 2021). Furthermore, the UV visible spectra of (GS and CS) CuO NPs revealed an absorption wavelength of 260 and 258 nm, respectively. Based on the absorption wavelength in the 200–350 nm range, the formation of CuO NPs was proven (Caroling et al. 2015; Akintelu et al. 2020). Moreover, the TEM images reflected that synthesized CuO NPs were spherical and had a uniform size of less than 50 nm. Their spherical shape and uniform size were previously observed by Jahan et al. (2021) and Fırat et al. (2022a, b). The presence of bright circular spots was detected in the present SAED profiles of (GS and CS) CuO NPs. This observation was proved by Bai et al. (2022), who stated that bright circular spots indicated the single-crystalline property of the CuO NPs. The SAED showed diffraction patterns directed towards (111), (202), (312), and (402) planes for GS-CuO NPs, and directed towards (111), (020), and (113) planes for CS-CuO NPs, which were in accordance with JCPDS card no. 00–041-0254, and can be indexed as a monoclinic CuO NPs shape. In proportion to SEM images, the prepared CuO NPs were relatively spherical. In addition, GS-CuO NPs were more aggregated than CS-CuO NPs, which was similar to the results reported by Siddiqui et al. (2021), who found that CuO NPs were relatively spherical and that the effect of the viscous nature of plant extract can cause CuO NPs to be aggregated. Besides, EDX results demonstrated that copper and oxygen were the primary elements in both synthesized NPs without any irrelevant peaks being detected, as Sabeena et al. (2022) revealed. The DLS and zeta potentials were 259.4 nm and − 10 mV, respectively, in water for GS-CuO NPs versus 218.3 nm and 19.5 mV, respectively, for CS-CuO NPs. The DLS results mean that the sizes of GS-CuO and CS-CuO NPs in water were > TEM measurements, remarkably in GS-CuO NPs; and this may be due to the aggregation of NPs. Similarly, GS-NPs showed a higher average of hydrodynamic diameter and aggregation, in comparison to CS-NPs (Anila et al. 2021). Additionally, the measured GS-CuO NPs zeta value was lower and near zero compared to that of CS-CuO NPs, which developed their high aggregation ability in water; this was in agreement with the SEM and DLS results. This was also confirmed by Jing et al. (2023), who stated that the positive and negative signs of the zeta potential indicate the surface charges of NPs, where NPs with a low zeta potential value (neutral or near zero) had a high tendency to aggregate.

### Oxidative stress biomarkers

The ability of harmful NPs to directly produce reactive oxygen species (ROS) has previously been described as a fundamental mechanism of fish toxicity (Srikanth et al. 2016; Fırat and Bozat 2019; Naeemi et al. 2020; Mahjoubian et al. 2021, 2023a). The detoxification capacity of fish can help eliminate their toxic effects (Xiang et al. 2020). However, the remaining balance will be destroyed and suffer oxidative damage if it is not quickly eliminated (Abdel-Khalek et al. 2020a). The liver acts as the primary tissue for the detoxification of toxic particles. However, gills are external tissues exposed to water persistently; and they are used to evaluate the fish's ability to rake different ROS (Ogunwole et al. 2021). GPx is a crucial enzyme detoxifying H_2_O_2_ (Temiz and Kargın 2022). Additionally, tissue-specific biomarker enzymes (CAT and SOD) are the first oxidative stress defenses (Kurian and Elumalai 2021). In this study, after adding (GS and CS) CuO NPs at both concentrations (25 and 50 mg/L) for 4 days, the enzyme parameters (GPx, SOD, and CAT) increased significantly in the examined tissues. This observation aligned with Shahzad et al. (2018), who demonstrated that the gill tissue SOD and CAT activities in *Oreochromis mossambicus* were significantly increased when treated with 0.5, 1, and 1.5 mg/L CuO NPs. The GPx elevation was also reported by Abd El-Atti et al. (2019) in the hepatic tissues of crayfish (*Procambarus clarkia*) after exposure to CuO NPs at different concentrations. The increase in responses of CAT and SOD accompanied a large increase in the activities of GPx, demonstrating that the SOD/CAT protection system could not remove the extreme ROS, and GPx contribution was required (Moussa et al. 2022). Abdel-Latif et al. (2021) noticed that fish were responding normally to protect themselves from the effects of CuO NPs-induced oxidative damage in their organs after observing that SOD, CAT, and GPx genes were significantly upregulated in the liver and gills of *O. niloticus* after exposure to 20 and 50 mg/L of CuO NPs. According to the current findings, the rise in the activities of GPx coincided with a steady decline in GSH concentrations of the studied tissues. This finding was consistent with that reported by Abdel-Khalek et al. (2015) regarding the tilapia tissues after exposure to CuO NPs. Moreover, the TBARS quantity was a helpful indicator for evaluating the health of cell membranes; and the LPO degree was used to gauge oxidative stress. The current study revealed that after being exposed to different concentrations of the synthesized NPs, the TBARS levels at the liver and gills gradually increased, which indicated the generation of oxidative damage and the formation of ROS that could cause severe harm to macromolecules such as DNA, proteins, and lipids (Ogunsuyi et al. 2019). Braz-Mota et al. (2018) found that the whole bodies of dwarf cichlid (*Apistogramma*) and cardinal tetra (*Paracheirodon*) species, which had been exposed to CuO NPs for 4 days, had high levels of TBARS; and they suggested that the antioxidants were not working well. Aziz and Abdullah (2023) also showed a significant rise in the levels of TBARS in the gill tissues of *Labeo rohita* exposed to CuO NPs. The oxidative stress biomarker responses were dose-dependent in both the synthesized CuO NPs groups, with the highest toxic effects observed when fish were exposed to a high sublethal concentration (50 mg/L). This observation agreed with Ganesan et al. (2016), Shahzad et al. (2018), and Aziz et al. (2022). Besides, this study demonstrated oxidative stress responses of GS-CuO NPs lower than those of CS-CuO NPs, which is comparable to Kurian and Elumalai (2021), who reported that the combination of plant-based extracts may have enhanced the antioxidant capability of *O. niloticus* exposed to GS-ZnO NPs.

### Copper bioaccumulation

Fish may take NPs from the water and store them in their active metabolite organs, such as the liver and gills. If they are exposed to certain levels of them, this could result in toxicological effects. The findings showed Cu content elevation in the examined tissues from all the groups. Moreover, the most significant values were detected in the groups exposed to the highest concentration of CS-CuO NPs (50 mg/L). This observation was in accordance with Riaz et al. (2020), who mentioned that sub-chronic exposure to CuO NPs, with an increase in dosage concentration, led to rising Cu accumulation in various tissues in *L. rohita*. The results also revealed that the liver, rather than the gills, had the highest bioaccumulation capacity for Cu, which was proved by Abdel-Khalek et al. (2020a). They stated that when nanoparticles build up, tissue-specific negative effects manifested because NPs might be disseminated equally across the tissues yet accumulate in distinct ways. According to Ling et al. (2016), metallothionein (MT) proteins often bound to primary trace metals, such as Cu and Zn, which might disturb the body’s internal equilibrium. The high rate of metallothionein synthesis might be related to the high hepatic NP buildup. Additionally, the synthesis of metal sulfur proteins in the liver tissues might be the origin of CuO liver abundance (Abdel-Khalek et al. 2020b). Due to the capacity of the gills to bind more NPs, preventing them from entering the body fluids, and the fact that they are constantly in contact with water, the accumulated CuO NPs were also seen in the gills (Shahzad et al. 2017; Aziz et al. 2022). The liver and gill BAF values showed the lowest entrance percentage in the groups exposed to GS-CuO NPs. This might be as a result of their higher tendency to aggregate in water, as observed in the present SEM, DLS, and zeta potential results. According to Jahan et al. (2017), NPs transform through aggregation, disintegration, and surface alteration by interacting with water components, which might modify their toxic effects and environmental liberation. Our findings also indicated that CuO NPs were most readily accumulated in the liver and gills of the fish groups that received 50 mg/L, where a greater level of tissue damage induced (confirmed by the alternations in levels of oxidative biomarkers and histological lesions in the examined tissues); this is in comparison to the groups that took the 25 mg/L concentration. Although the lower concentration showed greater uptake into the fish, compared to the 50 mg/L CuO NPs concentration in water, these lower uptakes in the higher concentrations may be due to the aggregation of NPs.

### Histological study

The histological analysis revealed that the CuO NP exposure altered the tissue level in the intended organs (liver and gills) in *O. niloticus*, supporting the observed changes in oxidative stress biomarkers and Cu bioaccumulation potency in these tissues. The current investigation showed that there had been notable deteriorations in the liver’s histoarchitecture, including central vein rupture, blood cell infiltration, congestion, vacuolization, pyknotic nuclei, and necrosis. These alterations were consistent with what Soliman et al. (2021) observed in *O. niloticus* after adding CuO NPs, which included vacuolization and pyknosis. Furthermore, hepatic histological changes, such as blood sinusoidal congestion, vacuolization in hepatocytes, infiltration, and necrosis, had previously been observed following *O. niloticus*’ exposure to CuO NPs (Abdel-Latif et al. 2021). According to Abdel-Khalek et al. (2022), the hepatic histological alterations might be caused by increased hepatocyte metabolic activity in response to CuO NP toxicity. Additionally, the significant vascular abnormalities in central vein rupture, blood cell infiltration, and blood congestion, resulting from CuO NPs’ impact on the endothelium of blood vessels, impeded the flow of blood in the veins, as stated by Goda et al. (2023). Blood congestion may indicate that the liver also recruited a large blood volume for CuO NPs detoxification (Miranda et al. 2016). The hepatic vacuolization might be caused by an imbalance in material production rates or excessive fat buildup (Ciji and Bijoy Nandan 2014). In fact, the liver cell degeneration and pyknotic nuclei in our results agreed with Abdel-Khalek et al. (2022), who noted that pyknotic nuclei were frequently a sign of oxidative damage to cell membranes. In addition, inhibition of an enzyme, rupture of the cellular membrane, problems with protein synthesis, and gluconeogenesis could all result in necrosis (Rabitto et al. 2005). Hepatocyte necrosis might also be brought on by ROS formation due to CuO NPs’ interaction with hepatic enzymes and other proteins (Choi et al. 2010). Gill histological changes, such as liver histology, are examined in our research and regarded as significant indicators of CuO NPs toxicity. Due to their exterior placement, gills are continually in contact with the environment. As a result, they are more susceptible to external stimuli. Similar to the current study, epithelial lifting, hyperplasia, lamellar fusion, epithelial layer rupture, severe edema, severe congestion, telangiectasis of the secondary lamellae, and necrosis were seen previously in *C. carpio* (Forouhar Vajargah et al. 2018) and *O. niloticus* (Abdel-Latif et al. 2021) treated with CuO NPs. According to Santos et al. (2021), epithelial lifting was one of the earliest histopathological reactions in fish gills, and it prolonged the travel time and distance for CuO NPs to reach the bloodstream. Capaldo et al. (2019) also stated that hyperplasia and lamellar fusion were protective mechanisms that reduced the respiratory surface (associated with CuO NPs) and its absorption value. These pointed to diminished gill function, which might result in an inadequate supply of oxygen, systemic hypoxia, respiratory disturbances, and fish death (Subashkumar and Selvanayagam 2014). According to Murali et al. (2018), decreased O_2_ uptake could cause blood vessel weakening, blood flow problems, congestion, and aneurysms. The branchial ^Na+/K+−ATPase’s^ suppression of ionic transport across the gill epithelium by CuO NPs, which implied a disturbance of osmotic control and subsequent osmotic imbalance, might be strongly related to the harmful effects of CuO NPs on gill edema (Al-Bairuty et al. 2013). Necrosis possibilities might rise as CuO NPs buildup grows, as previously observed by Almansour et al. (2017). Generally, the histological examinations in the liver and gills demonstrated that the severity of the alteration was most obvious in the groups exposed to CS-CuO NPs. Anila et al. (2021) also observed that CS-Pd NPs exhibited more tissue lesions than GS-Pd NPs in *D. rerio*, confirming our observation. The higher uptake percentage and bioaccumulation of the CS-CuO NPs might be responsible for the increased severity of observed lesions in the liver and gill tissues. The histological responses of the liver and gill tissues to CuO NP-induced toxicity in the current investigation were dose-dependent. Similar results were obtained from an earlier investigation, where *C. carpio* was subjected to the highest Cu NPs dose and displayed severe histological changes (Noureen et al. 2021).

## Conclusion

In this study, for the first time, GS-CuO NPs and CS-CuO NPs at different concentrations, as well as their nature in water and possible effects on different health biomarkers (oxidative stress, bioaccumulation of Cu, and histological effects) on the liver and gills of *O. niloticus*, were compared. Although GS-CuO and CS-CuO NPs are nearly similar in size and appearance, they differ in stability and behavior in water. The results showed that the GS-CuO NPs aggregated more in water, which might relate to the viscous nature of plant extracts and the surface charges of the NPs. According to the results of the comparative toxicity study conducted between both synthesized CuO NPs concentrations on *O. niloticus*, CS-CuO NPs in both concentrations induced more oxidative stress, exhibited higher accumulation in fish tissues, and caused severe histological alterations. Though GS-CuO NPs revealed toxic effects on *O. niloticus* fewer than those caused by CS-CuO NPs in the current study, they still have shown significant level of toxicity that is worthy of consideration. Given the current findings, more research is needed to evaluate additional GS-CuO NP features in water and their impact on various fish species. In addition, it is recommended to develop methods to ensure their safety in aquatic environments.

## Data Availability

The datasets used and/or analyzed during the current study are available from the corresponding author on reasonable request.
